# Haptic and visuo-haptic impairments for object recognition in children with autism spectrum disorder: focus on the sensory and multisensory processing dysfunctions

**DOI:** 10.1007/s00221-024-06855-2

**Published:** 2024-05-31

**Authors:** G. Purpura, S. Petri, R. Tancredi, F. Tinelli, S. Calderoni

**Affiliations:** 1 School of Medicine and Surgery, University of Milano Bicocca, Monza, Italy; 2 Unit for Visually Impaired People, Istituto Italiano di Tecnologia, Genova, Italy; 3 Department of Informatics, Bioengineering, Robotics and Systems Engineering (DIBRIS), Università degli Studi di Genova, Genoa, Italy; 4 Department of Developmental Neuroscience, IRCCS Fondazione Stella Maris, Pisa, Italy; 5 Department of Clinical and Experimental Medicine, University of Pisa, Via Roma 55, Pisa, 56126 Italy

**Keywords:** Autism spectrum disorder, Sensory processing, Multisensory integration, Visual processing, Haptic processing, Visuo-haptic transfer, Neurodevelopmental disorders, Children

## Abstract

Dysfunctions in sensory processing are widely described in individuals with autism spectrum disorder (ASD), although little is known about the developmental course and the impact of these difficulties on the learning processes during the preschool and school ages of ASD children. Specifically, as regards the interplay between visual and haptic information in ASD during developmental age, knowledge is very scarce and controversial. In this study, we investigated unimodal (visual and haptic) and cross-modal (visuo-haptic) processing skills aimed at object recognition through a behavioural paradigm already used in children with typical development (TD), with cerebral palsy and with peripheral visual impairments. Thirty-five children with ASD (age range: 5–11 years) and thirty-five age-matched and gender-matched typically developing peers were recruited. The procedure required participants to perform an object-recognition task relying on only the visual modality (black-and-white photographs), only the haptic modality (manipulation of real objects) and visuo-haptic transfer of these two types of information. Results are consistent with the idea that visuo-haptic transfer may be significantly worse in ASD children than in TD peers, leading to significant impairment in multisensory interactions for object recognition facilitation. Furthermore, ASD children tended to show a specific deficit in haptic information processing, while a similar trend of maturation of visual modality between the two groups is reported. This study adds to the current literature by suggesting that ASD differences in multisensory processes also regard visuo-haptic abilities necessary to identify and recognise objects of daily life.

## Introduction

Autism Spectrum Disorders (ASD) are a heterogeneous and complex group of neurodevelopmental conditions, described with a current prevalence of 1 in every 36 children aged 8 years (Maenner et al. 2023).

Although the most distinct symptoms of ASD have always been deficits in social communication and interaction, there have been indications of difficulties in processing sensory elements of the “non-social world” since Leo Kanner’s initial descriptions (Volkmar and McPartland 2014). However, it was only more recently that sensory dysfunctions were formally recognised as one of the core symptoms of ASD in the Diagnostic and Statistical Manual of Mental Disorders- 5th Edition (DSM-5), within the diagnostic criterium of restricted, repetitive patterns of behaviour, interests, or activities (APA 2013).

Specifically, the hypothesis of many sensory processing issues in children, adolescents and adults with ASD was widely described through the use of clinical tools/reports (Baranek et al. 2006; Chen et al. 2009; Leekam et al. 2007; Purpura et al. 2022; Valagussa et al. 2022) and more objective measures, including several paradigms of electrophysiology, psychophysics, and neuroimaging (Huang et al. 2022; Riva et al. 2022; Russo et al. 2008; Schoen et al. 2009; Spencer et al. 2000; Tavassoli et al. 2016; Turi et al. 2016).

The importance of this knowledge is gradually proving necessary to understand better the difficulties of ASD individuals in learning and interacting with the environment, even if the underlying mechanisms of the sensory dysfunctions are not so clear. In this conceptual framework, neuroanatomical and neurofunctional studies highlight altered connectivity in brain regions involved in low-level sensory processing in infants (Lewis et al. 2017) and toddlers with ASD (Chen et al. 2021) as possible neural underpinnings of sensory processing abnormalities and multisensory integration impairments frequently reported in ASD individuals since the early phases of development (Estes et al. 2015; Germani et al. 2014).

Notably, Baum and colleagues (Baum et al. 2015) summarised the five best-established theories that explain the enigma of ASD through the lens of sensory dysfunctions, concluding that several different components of these theories provide insights into ASD neurobiology, confirming the key role of sensory impairments in the altered functioning of this population. According to this view, sensory information represents the building blocks for the construction of higher mental functions. Therefore, the integration of these different sources of sensory information appears fundamental for the evolution of sensory processing towards the resultant sensory representations and socio-cognitive abilities. As a matter of fact, dysfunctions in sensory processing impact general functioning from the early periods of life (Butera et al. 2020), probably because differences to typical development are not limited to a single sensory modality but rather include multiple sensory systems such as vision, hearing, touch, proprioception, taste, and smell (Apicella et al. 2020; Kozou et al. 2018; Mansour et al. 2021; Muratori et al. 2017; Miguel et al. 2017; Shafer et al. 2021).

Nonetheless, research focused heavily on characteristics of sensory dysfunctions in adults and adolescents with ASD, while still little is known about the developmental course of sensory and multisensory processing in children with ASD and about the impact of these difficulties on the learning processes during preschool and school ages.

In particular, as regards the interaction between visual and haptic information in ASD during developmental age, knowledge is very scarce and controversial.

In 2012, Nakano et al. (Nakano et al. 2012) tested a group of adults with ASD in comparison with a control adult group through an experimental paradigm based on the “weak central coherence” theory (Frith 1989). Their findings suggested that individuals with ASD displayed superior abilities in a haptic-to-visual delayed shape-matching task in comparison with controls. According to the authors, the adults with ASD had a multimodal shape representation and haptic-to-visual information transfer more accurately than individuals without ASD (Nakano et al. 2012). Conversely, Poole et al. (Poole et al. 2017) suggested that adults with ASD showed statistically comparable performance to neurotypical subjects in processing visuo–haptic cues. Subsequently, the same research group confirmed that the ability to make size judgements using visual–haptic cues is similar for young and older adults with ASD. However, the process used differs according to age (Couth et al. 2019). More recently, Shafer and collaborators (2021) proposed a test of precision gripping to a group of adolescents with ASD in comparison with controls. ASD individuals demonstrated lower performance in integrating somatosensory feedback during visually guided manual motor behaviour, suggesting the presence of deficits in integrating multiple sources of sensory feedback to guide precision motor behaviour (Shafer et al. 2021). This aspect was also indicated by Ropar and collaborators (Ropar et al. 2018), while in preschoolers with ASD, Espenhahn and collaborators showed altered tactile perception using a psychophysical approach (Espenhahn et al. 2023).

Of note, research on this topic is lacking, and, to our knowledge, no studies currently highlight the cross-modal visuo-haptic sensory interaction for the recognition of objects or some elements of objects during manual activities in children with ASD.

In this light, this study aims to investigate visuo–haptic transfer abilities in preschool and school-aged children with ASD in comparison to matched controls with typical development (TD) using an experimental protocol already implemented and published by Purpura et al. (Purpura et al. 2018). Through this behavioural paradigm, it is possible to assess unisensory visual abilities, unisensory haptic abilities and multisensory visuo-haptic transfer for object recognition. Performance consistent with this model has been robustly replicated for visuo–haptic processing in children with cerebral palsy and in children with peripheral visual impairments (Purpura et al. 2019, 2021). We hypothesise that children with ASD show differences in the development of unisensory and multisensory visuo-haptic processes linked to object recognition compared to TD peers.

## Materials and methods

### Sampling

This study included a total of 70 pupils (48 M; 22 F; 5–11 years old age range). Out of the total sample, half of the children (*n* = 35) received a diagnosis of ASD and were recruited from the Division of Developmental Psychiatry of IRCCS Stella Maris Foundation in Pisa (Italy). They were evaluated in the Vision Laboratory of the same tertiary care university hospital between January 2019 and June 2022, according to the following criteria: (i) diagnosis of ASD performed by a multidisciplinary team, according to DSM-5 criteria; (ii) age between 4 and 11 years; (iii) total intelligence quotient > 70 at Wechsler Scales; (iv) absence of major sensory impairments in the child. The exclusion criteria were as follows: (i) children with genetic, neurological, or other psychiatric conditions; (ii) children with epilepsy or seizures controlled by pharmacotherapy; (iii) children with a deficit of stereopsis and visual acuity < 0.80 (decimal). The other half of the sample (*n* = 35) were term-born children with typical development (TD), matched for age and gender, recruited from a kindergarten and a primary school in Pisa (Italy). The inclusion criteria for this latter group were: (i) frequency of regular kindergarten or primary school without support teacher (the Italian law provides teacher support for children with developmental/clinical problems); (ii) no parent concern about child development, as indicated by a no-answer to a descriptive question in consent form; (iii) gestational age at birth ≥ 37 weeks. No TD child enrolled in the study had major ophthalmological or neuropsychiatric disorders. All children had an intelligence quotient > 15th percentile on Raven’s Progressive Matrices. Sample information is reported in Table 1.

**Table 1:** Socio-demographic information of study participants

	ASD (*n* = 35)	TYP (*n* = 35)
Gender (M, F)	24,11	24,11
Age (mean, SD) Age range	7.8 (1.9)	7.8 (1.9)
	5–11	5-10.8
Total IQ	98.1 (quotient)	69.8 (percentile)
ADOS-CSS	5.9 (1.7)	

*Abbreviation *M, male; F, female, SD, standard deviation; IQ, intellectual quotient; ADOS-CSS, calibrated severity scores at Autism Diagnostic Observation Schedule − 2

### Experimental design

We replicated the experimental design presented to typical preschool and school-aged children by Purpura and colleagues (Purpura et al. 2018) as we demonstrated that this design is effective in measuring visual, haptic and visual-haptic abilities regardless of the cognitive level (Purpura et al. 2018, 2019, 2021). The procedure required participants to perform an object-recognition task of familiar tools in everyday life by relying on unisensory (visual, haptic) or multisensory (visuo-haptic) inputs. In line with other already published studies (Bushnell and Baxt 1999; Martinovic et al. 2012), we selected 30 commonly everyday objects that should be familiar to children in the studied age groups (see Table 2) based on two main criteria. The first is related to the familiarity of objects in everyday life; indeed, the list includes toys, articles for personal hygiene, pieces of cutlery or household artefacts, school supplies, and so forth—that is, things that the children from 4 to 11 years of age had presumably held in their hands on many prior occasions, that probably had meaning for them, and for which they probably had conventional verbal labels. The second is related to the size of objects. Namely, we wanted to be sure that children could equally grasp and manipulate all the objects easily with one hand.

**Table 2:** A schematic view of the 30 objects, divided into three sets

SET A	SET B	SET C
Ring	Coffee cup	Hair elastic
Die	Little sponge	Button
Little ball	Eraser	Screw
Dummy (pacifier)	Paper clip	Comb
Teaspoon	Little toy car	Little brush
Clothes peg	Bracelet	Battery
Coin	Key	Pen cap
Little candle	Little plastic tube (toothpaste)	Pencil sharpener
Cork (bottle cap)	Little toy bear	Building block (Lego-like)
Little dessert fork	Pencil	Children’s scissors

The objects were randomised into three sets of 10 objects each. Following pseudorandom criteria, all three sets were administered to the children, each assigned to a different sensory modality. To guarantee an equal presentation of each set in the visual, haptic, and visuo-haptic modalities, six combinations of sets were arranged for the children (for more information, see the previous studies). A six-second time restriction was set for the sensory exploration of each item based on two main considerations. Firstly, this time limit is used in several standardised protocols within the clinical settings to compare the performances of typically developing children and children affected by different developmental disorders. For instance, several clinical and experimental studies performed on children (Desmarais et al. 2017; Giannopulu et al. 2008; Gori et al. 2008a; Jovanovic and Drewing 2014; Kalagher and Jones 2011) used a time restriction for sensory explorations (visual and/or tactile) of stimuli (objects or geometrical shape) from 5-to-less seconds. Similarly, Morrongiello et al. (Morrongiello et al. 1994) found that a response latency of 6 s is sufficient for tactile exploration and recognition of normal-sized objects in sighted and blind children. Secondly, preliminary multiple evaluations collected for this purpose (unpublished) suggested that this time limit was sufficient for adults and children with and without developmental disorders to be confident in performing an objects-recognition task in all three modalities (visual, haptic and visuo-haptic). After this time (6 s), participants were encouraged to answer, even when they found the task difficult or were loath to respond. In all tasks, the requested response was the naming of the object. Verbal response times during the test phase were unrestricted. Eventual phonologic errors were not considered if the pronounced word appeared comprehensible. Synonymous terms were considered as corrected. We also included as correct the answers of children who were not able to indicate the correct name of the object, but only its exact function (for example, if the child is not able to say the term “coffee cup”, but he/she says “for drinking coffee” or if the child recognises the little tube but he/she says “tube of glue” instead “of toothpaste”). Children (ASD and TD) with refractive errors used their usual prescription spectacles. As verified in our previous study (Purpura et al. 2018), the protocol was easy and rapid to administer (15–20 min); the stimulus aroused the children’s interest, which helped keep their attention, making a good level of collaboration. For ASD children, the administration was performed inside of the hospital routine care. Before administering the battery, we obtained parents’ written informed consent for their child’s participation in the study. The study was conducted in accordance with the Declaration of Helsinki.

#### Visual object recognition

**Fig. 1:**
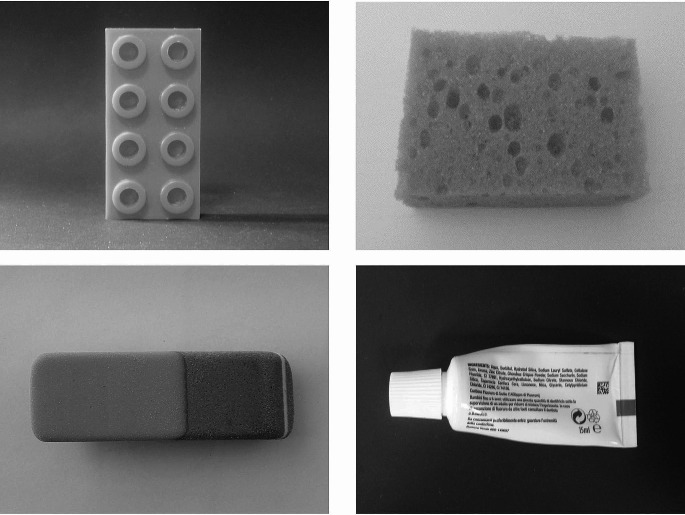
Examples of the stimuli used for the recognition tasks

In the visual object recognition task (V-ORT), participants were presented with black-and-white photographs (12 × 9.2 cm) of real objects from the usual viewer perspective, all with the same background and environmental context. (see Fig. 1). Each photograph was removed after the six-second exploration time, and children were asked to report the name of the object depicted. The experimenter verbally instructed participants as follows: “Tell me the name of the object you just saw in the picture”.

#### Haptic object recognition

In the haptic object recognition task (H-ORT), participants were asked to recognise objects through tactile exploration alone with no visual input. Participants held their dominant hand inside a box that contained the target object and prevented them from looking at the object itself. After the six-second exploration time, the object was removed, and participants were asked to report the name of the object they had previously touched. The experimenter verbally instructed participants, “Tell me the name of the object you just touched within the box.”

#### Visuo-Haptic object recognition

In the visuo-haptic object recognition task (VH-ORT), participants were asked to recognise objects with simultaneous visual and haptic inputs. Specifically, children explored the object placed within the box with their dominant hand and simultaneously viewed four different objects (including the one inside the box) depicted in black-and-white photographs (size 12 × 9.2 cm) placed in front of them. Among the photographs presented, three served as distractor stimuli, since they represented objects semantically similar or similar in shape to the target object. The visuo-haptic task was designed in order to create a condition in which enriched information about the structure of an object is presented: indeed, as suggested by some authors (Lacey and Campbell 2006) touch might preferentially convey three-dimensional structural information, while vision might preferentially convey two-dimensional geometric information. As in the unisensory only-visual and only-haptic tasks, participants were asked to report the name of the object they explored both by vision and touch after the six-second exploration time. Pointing responses were not considered. The experimenter verbally instructed participants as follows: “Tell me the name of the object you just touched within this box, considering the visual picture you see in front of you”.

### Scoring

As discussed in the previous paragraph, the list of 30 items to be recognised was split into three sets, each consisting of 10 objects per sensory modality. A score of 1 was given for each object the child successfully identified, and 0 if the subject either failed to recognise it correctly or did not respond. In this way, the subject could score a minimum of 0 and a maximum of 10 for each sensory modality.

### Statistical analysis

All analyses were carried out using IBM SPSS Statistics 28.0.0 software. A p-value below 0.05 was interpreted as significant. Although the Shapiro–Wilk test for normality suggested the use of a non-parametric statistical approach because most variables showed a non-normal distribution, we opted for using a mixed approach to better study the interaction effects. First, the results between the two groups (ASD and TD) across the three different sensory modalities (V-ORT, H-ORT, and VH-ORT) were compared through the non-parametric Mann–Whitney Test. Subsequently, a mixed-design ANOVA analysis was carried out to perform a confirmatory analysis, with sensory modalities (V-ORT, H-ORT, VH-ORT) as a repeated-measure factor and group (ASD and TD) as a between-participant factor. Post-hoc tests (Bonferroni) were performed. Secondly, we used the Wilcoxon Test to compare scores on V-ORT, H-ORT, and VH-ORT separately within the ASD and TD groups.

Moreover, the Mann-Whitney Test (with Bonferroni correction) was performed to assess the correct answers of ASD and TD children across three age sub-groups (Group A: second and third kindergarten classes, 4–5 years; Group B: first, second, and third primary school classes, 6–8 years; Group C: fourth and fifth primary school classes, 9–11 years) and the Wilcoxon Test (with Bonferroni correction) was used to compare scores on V-ORT, H-ORT, and VH-ORT within the three age groups, separately for ASD and TD groups.

Partial non-parametric correlation analysis (Spearman Test), controlled for gender, was performed between the scores at different tasks and ages, divided into two groups (ASD and TD). Finally, in the ASD Group, a two-tails bivariate non-parametric correlation test (Spearman Test) between the scores at different tasks and some clinical data (IQ and ADOS scores) was also carried out.

## Results

The mean values of the correct answers obtained in the three sensory modalities show different score distributions in the two groups (ASD Group = Mean V-ORT: 8.23, SD: 1.4; Mean H-ORT: 6.3, SD: 2.0; Mean VH-ORT: 8.5, SD 1.2; TD Group = Mean V-ORT: 8.7, SD = 1.0; Mean H-ORT: 8.0, SD = 1.5; Mean VH-ORT: 9.6, SD 0.5) (see Table 3).

**Table 3:** Mean correct answers of the two groups in visual, haptic and visuo-haptic recognition tasks and p-value at Mann Withney U Test

	ASD (*n* = 35)	TYP (*n* = 35)	*p*-value
V-ORT	8.2 (1.4)	8.7 (1.0)	0.160
H-ORT	6.3 (2.0)	8.0 (1.5)	< 0.001
VH-ORT	8.5 (1.2)	9.0 (0.5)	< 0.001

**Fig. 2:**
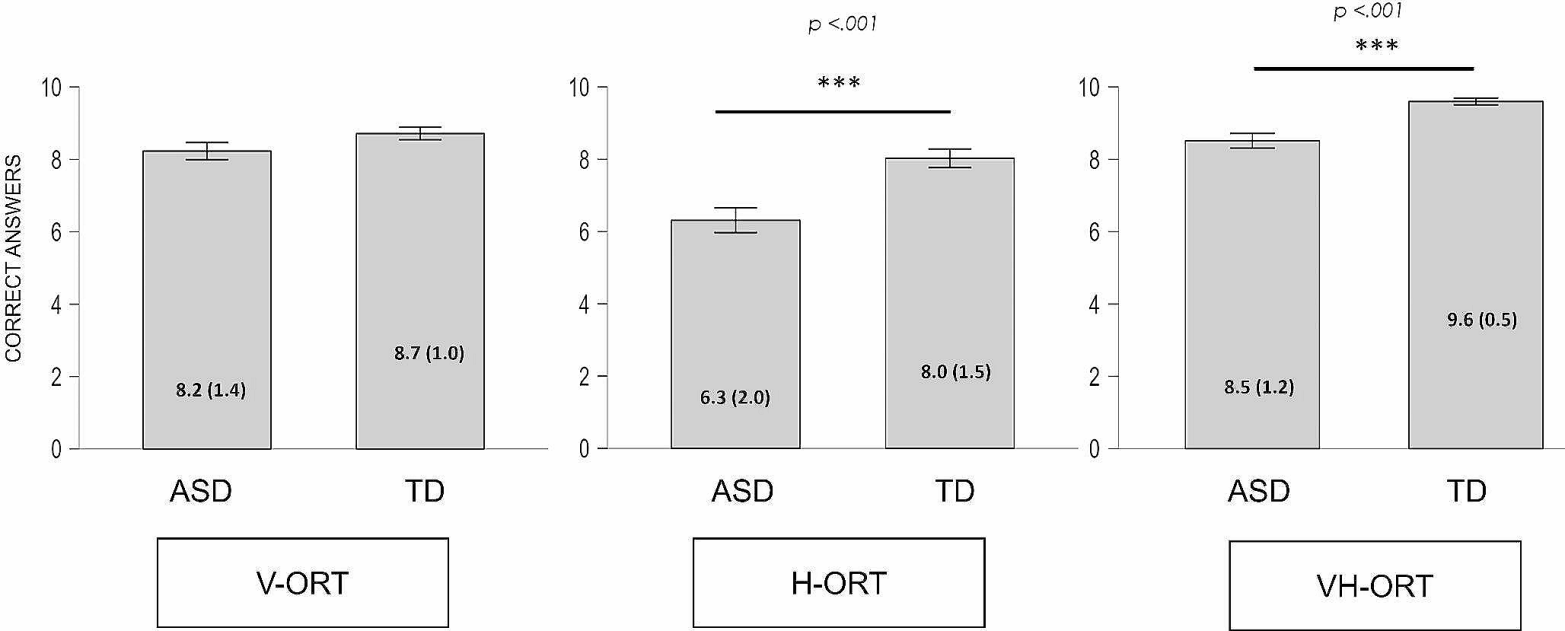
Mean correct answers of the two groups in visual, haptic and visuo-haptic recognition tasks and p-values at Mann Withney U Test

The non-parametric Mann –Whitney Test between the ASD group and the TD group indicated statistically significant differences in the number of correct answers on H-ORT (W = 296.000; *p* < 0.001) and VH-ORT (W = 278.500; *p* < 0.001), while there were no differences in V-ORT (W = 497.000; *p* = 0.160) (see Fig. 2).

After, the mixed-design ANOVA analysis was carried out with sensory modalities (V-ORT, H-ORT, VH-ORT) as a repeated-measure factor and group (ASD and TD) as a between-participant factor yielded a significant effect of sensory modalities (*F (2) = 54.167*, *p* < 0.001) and group (*F (1*) = 19.894, *p* < 0.001) on task performance. Bonferroni’s Post Hoc test confirmed the main effect of sensory modality (V-ORT vs. H-ORT: mean differences 1.300, t = 7.010, *p* < 0.001; V-ORT vs. VH-ORT: mean differences − 0.586, t = -3.158, *p* = 0.006; H-ORT vs. VH-ORT: mean differences − 1.886, t = -10.168, *p* < 0.001), but also the main effect of the group (ASD vs. TD: mean difference − 1.095, t= -4.460, *p* < 0.001). These main effects were qualified by a significant interaction between sensory modalities and group (*F (2) = 5.487*, *p* = 0.005). The comparison between the two groups through the Bonferroni Post-hoc Test was significantly different in haptic and visuo-haptic tasks (H-ORT: mean difference − 1.714, t= -5.262 *p* < 0.001; VH-ORT: mean difference − 1.086, t= -3.332, *p* = 0.016), but not in the visual one (V-ORT: mean difference − 0.486, t= -1.491 *p* = 1.000).

Moreover, Bonferroni’s Post Hoc tests showed that multisensory visuo-haptic skills were better than both unisensory skills in the TD group but not in the ASD group. As a matter of fact, significant differences between V-ORT and H-ORT (mean differences 1.914, t = 7.299, *p* < 0.001) and between H-ORT and VH-ORT (mean differences − 2.200, t = -8.388, *p* < 0.001) were found in the ASD group, but not between V-ORT and VH-ORT (mean differences − 0.286, t = -1.089, *p* = 1.000). Conversely, the analysis highlighted significant differences between V-ORT and VH-ORT (mean differences − 0.886, t = -3.377, *p* = 0.014) and between H-ORT and VH-ORT (mean differences − 1.571, t = -5.992, *p* < 0.001) in the TD group, but not between V-ORT and H-ORT (mean differences 0.686, t = 2.614, *p* = 0.149).

**Fig. 3:**
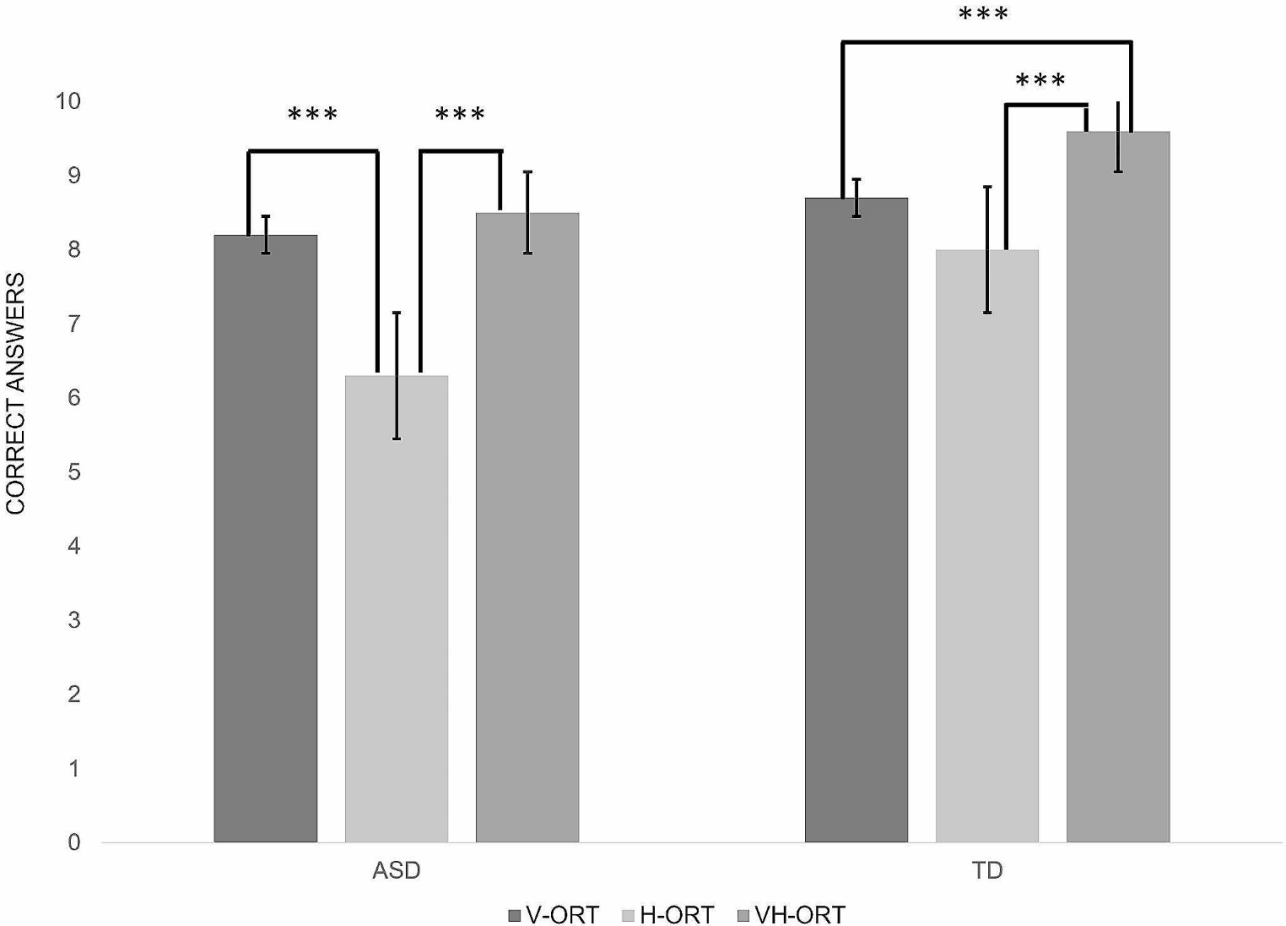
Differences in the number of correct answers in the three sensory conditions across the two sample groups (ASD group vs. TD group). The asterisks indicate a significant difference between conditions: ***p ≤ 0.005

Also for this case, findings were confirmed by non-parametric analysis. Within the ASD group, the Wilcoxon Test (Bonferroni corrected α = 0.016 [.05/3]) revealed significant differences in the number of correct answers between V-ORT and H-ORT (z = − 4.703; *p* < 0.001) and between H-ORT and VH-ORT (z = − 4.642; *p* < 0.001), but not between V-ORT and VH-ORT (z = -1.185, *p* = 0.236) (see Fig. 3). These results are different from those obtained by the Wilcoxon Test (Bonferroni corrected α = 0.016 [.05/3]) on the TD sample (see Fig. 3), which showed significant differences between V-ORT and VH-ORT and between H-ORT and VH-ORT, but not between V-ORT and H-ORT (V-ORT vs H-ORT: z = − 2.379, *p* = 0.017; V-ORT vs VH-ORT: z = − 3.710, *p* < 0.001; H-ORT vs VH-ORT: z = − 4.122, *p* < 0.001).”

**Fig. 4:**
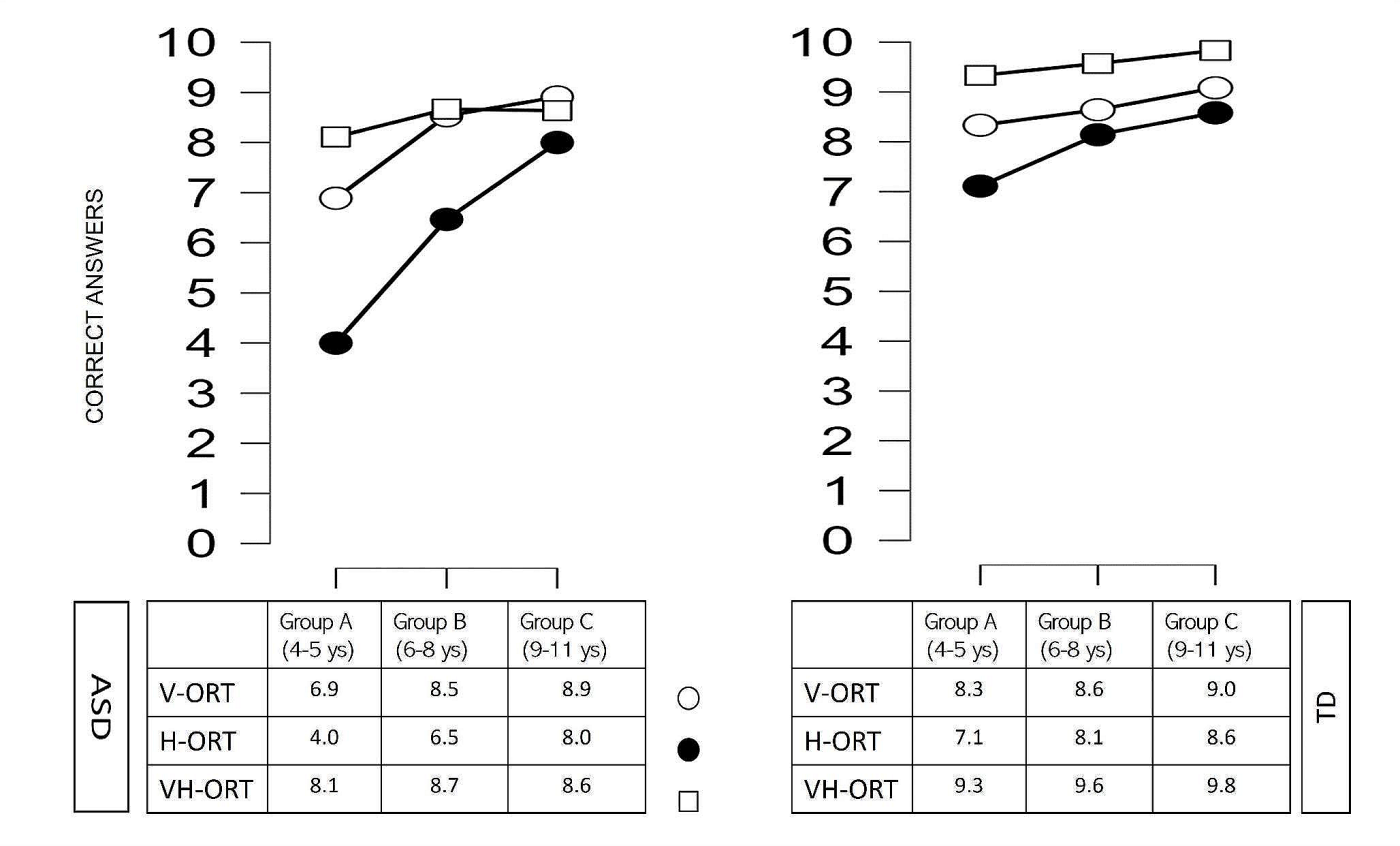
Developmental trend in the three sensory conditions both in the ASD group and in the TD group. The circles indicate the unimodal conditions tasks (white circles for the visual recognition task and black circles for the haptic recognition task), while the squares indicate the bimodal condition task (visuo-haptic task). (Group A: second and third kindergarten classes, 4–5 years; Group B: first, second, and third primary school classes, 6–8 years; Group C: fourth and fifth primary school classes, 9–11 years)

Based on the data from the three age groups (see Purpura et al. 2018), a different trend of development in unisensory and multisensory modalities of ASD children was evident compared to TD children (see Fig. 4). About Group A (children of second and third kindergarten class – from 4,0 to 5,11 years of age; *n* = 9 for group), the Mann-Whitney Test showed significantly higher scores in TD children with respect to ASD children only in H-ORT (V-ORT: z = − 2.234; *p* = 0.026, H-ORT: z = − 2.685, *p* = 0.007; VH-ORT: z = − 2,168, *p* = 0.030, Bonferroni corrected α = 0.016 [0.05/3]). Similarly, as regards Group B (children of first, second and third primary school classes – from 6,0 to 8,11 years of age; *n* = 14 for group), the significant differences between the two samples were present in the haptic condition (V-ORT: z = − 0.364, *p* = 0.716; H-ORT: z = − 3.377, *p* < 0.0.001, VH-ORT: z = − 2.285, *p* = 0.022, Bonferroni corrected α = 0.016 [0.05/3]). Finally, in Group C (children of fourth and fifth primary school classes – from 9,0 to 11,0 years of age; *n* = 12 for group), the difference between ASD and TD remained significant only for VH-ORT (V-ORT: z = − 0.521, *p* = 0.603; H-ORT: z = − 1.366, *p* = 0.172; VH-ORT: z = − 2.880, *p* = 0.004, Bonferroni corrected α = 0.016 [0.05/3]).

Furthermore, regarding the ASD children, the Wilcoxon Test (Bonferroni corrected α = 0.016 [0.05/3]) showed in younger children of Group A and B, significant differences between visual and haptic tasks and between haptic and visuo-haptic tasks (Group A: V-ORT vs. H-ORT- z = -2.585, *p* = 0.010; H-ORT vs. VH-ORT - z = -2.680, *p* = 0.007; V-ORT vs. VH-ORT - z = -1.982, *p* = 0.047; Group B: V-ORT vs. H-ORT - z = -3.400, *p* < 0.001; H-ORT vs. VH-ORT - z = -3.438, *p* < 0.001; V-ORT vs. VH-ORT - z = -0.359, *p* = 0.719), while in the older children of Group C, no significant differences were found (Group C: V-ORT vs. H-ORT - z = -1.995, *p* = 0.046; H-ORT vs. VH-ORT - z = -1.308, *p* = 0.191; V-ORT vs. VH-ORT - z = -0.551, *p* = 0.582). By contrast, regarding the TD children, no differences were found in younger children of Group A (Group A: V-ORT vs. H-ORT- z = -2.050, *p* = 0.040; H-ORT vs. VH-ORT - z = -2.121, *p* = 0.034; V-ORT vs. VH-ORT - z = -2.165, *p* = 0.030) and in older children of Group C (Group C: V-ORT vs. H-ORT - z = -1.066, *p* = 0.286; H-ORT vs. VH-ORT - z = -2.354, *p* = 0.019; V-ORT vs. VH-ORT - z = -1.983, *p* = 0.047), while a significant difference between haptic and visuo-haptic tasks was found in Group B (Group B: V-ORT vs. H-ORT - z = -1.090, *p* = 0.276; H-ORT vs. VH-ORT - z = -2.836, *p* = 0.005; V-ORT vs. VH-ORT - z = -2.292, *p* = 0.022).

Finally, partial correlation analysis (Spearman Test), controlled for gender, shows, in the ASD group, a significant correlation between age and the scores at V-ORT (rho = 0.507, p-value = 0.002) and between age and the score at H-ORT (rho = 0.698, p-value < 0.001), but not between age and the scores at VH-ORT (rho = 0.224, p-value = 0.202). On the contrary, in the TD group, a significant correlation between age and the scores at H-ORT (rho = 0.400, p-value = 0.019) and between age and VH-ORT (rho = 0.341, p-value = 0.048) was found, but not between age and the scores at V-ORT (rho = 0.247, p-value = 0.158). According to the Spearman Test, in the ASD group, neither a significant correlation between ADOS score and the three sensory conditions nor between IQ and the three sensory conditions was found. For a more detailed description of findings obtained with TD children, see Purpura et al. 2018.

## Discussion

To the best of our knowledge, this is the first study that investigated unimodal sensory processing abilities of visual and haptic stimuli and bimodal visuo-haptic transfer abilities in ASD children through object recognition tasks. Although sensory dysfunctions in visuo-haptic integration of ASD children are documented in scientific literature, in all cases, these hypotheses were supported mainly by data from questionnaires or interviews with caregivers (Purpura et al. 2022; Rogers et al. 2003; Simpson et al. 2019; Ting 2013), in the absence of the child’s direct evaluation. For our study, we instead utilised a behavioural paradigm that had already given robust and interesting results about unimodal and cross-modal skills in children with typical development (Purpura et al. 2018), in children with congenital brain lesions (Purpura et al. 2019) and in children with visual disorders (Purpura et al. 2021).

The first main finding of the present study is that TD and ASD children differ in the developmental refinement of haptic and visuo-haptic perceptual abilities. This finding is not surprising since several studies reported other atypical sensory integration processing (e.g., audio-visual integration processing) in children, adolescents, and adults with ASD (for a recent review, see Beker et al. 2018). Multisensory processes consist of the ability of the brain to integrate information from different sensory channels to speed and enhance its ability to detect, locate, and identify external events, to disambiguate potentially confusing signals, as well as to organise the higher-order and behavioural processes necessary to deal with the surrounding environment (Perrault et al. 2012). Indeed, when cross-modal stimuli are spatially and temporally congruent, they elicit more vigorous responses and produce enhanced neural and behavioural responses (Stein et al. 2020). The developmental course of these processes is very long and complex: Gori and collaborators (Gori et al. 2008b) showed that before 8 years of age, haptic information dominates for size discrimination tasks, while vision dominates for orientation discriminations. According to these authors, only after 8 years of age does the integration of these two types of information appear to become statistically optimal, like adults. Hence, perceptual systems need constant recalibration during development through sensory experiences (Gori et al. 2008b). Despite this, multisensory facilitation is already possible from five years of age in typical development, both for recognition of geometrical shapes (Gori et al. 2024) and for objects of daily life (Purpura et al. 2018), although this ability is still immature at this age. This facilitation might additionally rely upon the truth that sensorimotor integration is an essential element of item recognition, on account that moving the object creates an active exploration inside the experience that the kids organise during their hand moves relative to the objects and of the object’s movement itself (Sciutti and Sandini 2022). According to our data, within-group analyses suggested that significant multisensory interactions for object recognition facilitation did not occur in ASD children compared to the TD group. Indeed, significantly lower performance between the two groups in VH-ORT was evident both considering the whole sample and considering only the older children’s groups (9–11 years of age). Specifically, a light, not statistically significant decrease in VH-ORT scores in this age group compared to the sample of 6-8-year-old children was found in the ASD population. This finding is in line with previous data in which individuals with ASD reported considerable effects of visual interferences on tactile judgement about their hands (Wada et al. 2021).

The second main finding of this study is that visuo-haptic transfer appears suboptimal in the ASD group because it relies mainly on visual information. In fact, ASD children reported a similar trend of maturation in visual modality as TD children, while a specific deficit of the haptic one was found. In agreement with our previous study (Purpura et al. 2018), visual recognition abilities are generally better than haptic recognition abilities both in the ASD group and in the TD group, although in ASD children, tactile processing appears notably weaker and seems to impact on the recognition of objects in daily life. Thus, this specific difficulty may not permit an appropriate interaction of haptic information with visual information, ultimately causing an altered transfer of cross-modal information. This finding is confirmed by comparison analysis between ASD and TD children also when divided into age groups. Indeed, the difference between ASD and TD children is evident in haptic processing until 8 years, while it tends to disappear from 9 years of age when the differences in functioning during cross-modal tasks begin to become clearer. This finding is consistent with the suggestions of Baum et al. (Baum et al. 2015), which affirmed that processing deficits within a sensory modality will undoubtedly result in processing changes when analysing multisensory function. Moreover, alteration in tactile information processing has already been described in children with ASD using tactile threshold detection tasks (Tavassoli et al. 2016) and vibrotactile tasks (Espenhahn et al. 2023; Puts et al. 2014).

This is also in line with the findings suggesting that typically object recognition by touch is much slower than by vision and that haptically presented objects usually require several seconds of exploration before being named (Craddock and Lawson 2008, 2009; Lacey and Campbell 2006). For this reason, some authors adopt a 1:2 ratio for the time of exploration in visual and haptic modalities (Lacey et al. 2011). In this sense, the same restriction time in visual and haptic tasks of our experiment (six seconds) may influence results about ASD children. Actually, our behavioural data confirmed that there are no differences between the two groups in visual processing, while in haptic processing, with an equal time, ASD children show lower capabilities than TD children. Moreover, there are no significant differences between the V-ORT score and the H-ORT score in TD children, suggesting a specific deficit in the haptic processing of ASD children.

The last main finding of the present study is that the significantly worse performance in the haptic task but not in the visual task of ASD children in comparison to TD children is the opposite functioning that occurred in children with low vision (Purpura et al. 2021). Indeed, in children with visual impairment, a deficit of visual recognition abilities in comparison to haptic recognition abilities did not permit an appropriate cross-modal calibration between vision and touch for the visuo-haptic recognition task. Therefore, in our sample of ASD children, during the visuo-haptic transfer task, the constituent unisensory stimuli cannot evoke a better response than that elicited by the visual task alone, according to the principle of inverse effectiveness. Indeed, as described by Stein and colleagues (Stein et al. 2014), multisensory enhancement is defined as a response to a cross-modal stimulus that exceeds the response to either of its modality-specific components. According to the principle of the inverse effectiveness of multisensory integration, this enhancement strongly increases for poorly perceptible congruent unisensory signals, i.e. as the responsiveness to individual sensory stimuli decreases, the strength of multisensory integration increases. However, at the same time, if cross-modal stimuli are disparate (for example, because of very different intensities), these are more likely to belong to unrelated or competing events and will either fail to interact or will interact competitively, thereby producing a response depression (degraded), and not enhancement. This is also suggested by the different trends of maturation during school age regarding unimodal and cross-modal abilities in the two groups since a specific developmental delay of haptic processing in ASD children is evident, which consequently impacts on cross-modal visual-haptic maturation.

Given the fact that multisensory integration represents a key building block in the construction of higher-order cognitive representation, a specific interference in visuo-haptic transfer may be linked to the reduced and atypical object exploration, contributing to the peculiar strategies in the use of objects of ASD children from the early periods of life (Ozonoff et al. 2008). As a matter of fact, the presence of some early atypicalities of sensory-motor development, such as a higher rate and a larger inventory of repetitive/stereotyped movements both with and without objects, is one of the most predictive symptoms of ASD during the first year of life (Elison et al. 2014; Miller et al. 2021; Posar and Visconti 2022; Purpura et al. 2017). In this regard, Kaur and colleagues observed significant differences in object exploration skills of ASD at-risk infants from 6 to 15 months (Kaur et al. 2015). These authors highlighted that at-risk infants showed similar but delayed developmental trajectories in exploratory behaviours compared to low-risk infants and that grasping and manipulation delays in at-risk children seemed to be attributable to specific object properties. According to these findings, specific red flags for atypical object exploration through the observation of tactile skills and reduced manual object exploration from the first years of life could be accurately considered for the assessment finalised for early detection of ASD in at-risk children. This aspect could have important implications for planning sensory and object-based interventions for children with ASD or at risk of ASD. Promoting early object interactions within a multisensory educative context could also have a valuable role in promoting a better functional adaptation to the environment of these children in the subsequent years. This point is critical because of the importance of different or unusual perceptual sensitivities in the daily lives of ASD individuals. Chamak and collaborators (Chamak et al. 2008) reported personal experiences of adults with ASD and compared them to scientific and medical knowledge and representations. Their results suggested that all ASD individuals attributed a key role in their behaviour to their unusual perceptual sensitivities, and all of them also pointed out that a different way of information processing represented the core symptom of their condition. These results offer important insights into the sensory special needs of children and adults with ASD.

## Limitations

Our study presents some limitations, here briefly discussed to orient future investigations on the same research topic. The first limitation entails the time limit imposed for the sensory exploration of objects before the recognition request. Some studies suggest that object recognition by touch is typically much slower than by vision and that haptically presented objects might require several seconds of exploration before being recognised (Craddock and Lawson 2008, 2009; Lacey and Campbell 2006). This has led to the adoption of a task procedure in which the time given to haptically explore objects is wider than the time given to visually explore the same object, as in studies adopting a 1:2 ratio for the time of exploration in visual and haptic modalities (Lacey et al. 2011). However, in the present study, we decided to keep the time limit equal (six seconds) for both visual and haptic exploration in order to directly compare the two modalities in terms of perceptual readiness and to directly compare the two groups in terms of perceptual performance. Our findings indicated that the six-second time limit is sufficient for both groups to perform the only-visual task condition and is also sufficient for the TD (but not for the ASD) group to perform the only-haptic task condition. This result seems to suggest that the poorer haptic performance of the ASD group is not related to the time limit imposed by the protocol but rather to a specific deficit in haptic processing.

The second limitation of the present work entails the use of photographs as visual stimuli. We decided to present black-and-white photographs of real objects and not drawings or the real objects themselves because photographs minimise the facilitatory concurrent effect of other sensory information (e.g. texture, colour) and their use might prevent automatic cross-modal processing (Snow and Culham 2021). Also, the choice is in line with neural data showing that the cortical networks involved in the recognition of familiar objects are the same for both vision and haptics modalities, independently of the fact that stimuli consist of real objects or photographs (Martinovic et al. 2012). Finally, other published studies used similar procedures with realistic photographs on the grey scale to study visual-haptic recognition (Jao et al. 2015; Joanne Jao et al. 2014). As a matter of fact, our findings reveal that using photographs as visual stimuli did not disadvantage the ASD group compared to the TD group. Furthermore, no performance differences have been reported between the two groups in the V-ORT, and scores in vision/photographs and haptics/real objects were equal in the TD group. Although there is room for improvement in our procedure, these data sustain an excellent capacity to reveal sensory processing alterations in children.

The third limitation of the present work is that participants were required to recognise objects in different ways when performing the unisensory and multisensory task conditions. Indeed, while in the unisensory conditions, they were asked to name the perceived object, in the multisensory condition, they had before to distinguish the target object among distractors and then name it. Moreover, in this case, there was an absence of a short delay between the presentation of the visual and haptic information in the visual-haptic transfer task since the two sensory clues were proposed simultaneously to facilitate multisensory enhancement. In this way, identifying the direction of sensory transfer (vision to touch vs. touch to vision) appears difficult. Indeed, a delayed matching-to-sample procedure with explicit memory demand could be used in future investigations to understand better the nature of multisensory defects for object recognition in ASD children.

A last limitation of the present work is the limited sample of participants enrolled, which does not permit a more accurate and detailed analysis within the two groups based on age. We then used non-parametric comparisons and partial correlation analysis to suggest that ASD and TD children may have different trends of maturation during school age regarding unimodal and cross-modal abilities. In particular, a specific developmental delay of haptic processing in ASD children is evident, which consequently impacts also on the cross-modal visuo-haptic maturation. Further studies with a larger sample of ASD pre-schoolers and schoolers could be helpful to shed light on this point.

## Conclusion

The goal of the present study was to compare the visual, haptic, and visuo-haptic transfer abilities for object recognition in ASD and TD children during the preschool and school years. Importantly, our study adds to the current literature by suggesting that group differences in multisensory processes already described in ASD individuals may also regard visuo-haptic abilities necessary to identify and recognise objects of daily life.

Although this study had some limitations, including a lack of more objective measures (using, for example, different electrophysiological and neuroimaging techniques), several insights can be highlighted that underline its novelty. Our results suggest that ASD children demonstrate a delay in haptic abilities for object recognition. At the same time, vision matures similarly to TD children and tends to dominate for the recognition of objects. Consequently, atypical abilities in visuo-haptic transfer of information in the group of ASD children are evident, and this deficit of cross-modal calibration appears to persist over time. As the data were collected through a customised behavioural battery, future studies could benefit from integrating complementary information derived from psychophysics or electrophysiological measures.

In conclusion, based on these insights, improvement of early rehabilitation programmes for children with ASD to guarantee early adequate exposure to cross-modal experiences is highly recommended if founded on ecological and evidence-based approaches.

## Data Availability

The data supporting this study’s findings are available from the corresponding authors upon request.

## References

[CR1] APA (2013) American Psychiatric Association, 2013. Diagnostic and statistical manual of mental disorders (5th ed.). In *American Journal of Psychiatry*

[CR2] Apicella F, Costanzo V, Purpura G (2020) Are early visual behavior impairments involved in the onset of autism spectrum disorders? Insights for early diagnosis and intervention. Eur J Pediatrics 179(2). 10.1007/s00431-019-03562-x 10.1007/s00431-019-03562-x 31901981

[CR3] Baranek GT, David FJ, Poe MD, Stone WL, Watson LR (2006). Sensory experiences Questionnaire: discriminating sensory features in young children with autism, developmental delays, and typical development. J Child Psychol Psychiatry.

[CR4] Baum SH, Stevenson RA, Wallace MT (2015). Behavioral, perceptual, and neural alterations in sensory and multisensory function in autism spectrum disorder. Prog Neurobiol.

[CR5] Beker S, Foxe JJ, Molholm S (2018). Ripe for solution: delayed development of multisensory processing in autism and its remediation. Neurosci Biobehavioral Reviews.

[CR6] Bushnell EW, Baxt C (1999). Children’s haptic and cross-modal recognition with familiar and unfamiliar objects. J Exp Psychol Hum Percept Perform.

[CR7] Butera C, Ring P, Sideris J, Jayashankar A, Kilroy E, Harrison L, Sharon C, Aziz-Zadeh L (2020). Impact of sensory Processing on School Performance outcomes in High Functioning individuals with Autism Spectrum Disorder. Mind Brain Educ.

[CR8] Chamak B, Bonniau B, Jaunay E, Cohen D (2008). What can we learn about autism from autistic persons?. Psychother Psychosom.

[CR10] Chen YH, Rodgers J, McConachie H (2009). Restricted and repetitive behaviours, sensory processing and cognitive style in children with autism spectrum disorders. J Autism Dev Disord.

[CR9] Chen B, Linke A, Olson L, Ibarra C, Reynolds S, Müller R, Kinnear M, Fishman I (2021). Greater functional connectivity between sensory networks is related to symptom severity in toddlers with autism spectrum disorder. J Child Psychol Psychiatry.

[CR11] Couth S, Poole D, Gowen E, Champion RA, Warren PA, Poliakoff E (2019). The Effect of Ageing on Optimal Integration of Conflicting and Non-conflicting Visual-Haptic Stimuli. Multisensory Res.

[CR12] Craddock M, Lawson R (2008). Repetition priming and the haptic recognition of familiar and unfamiliar objects. Percept Psychophys.

[CR13] Craddock M, Lawson R (2009). Do Left and Right Matter for Haptic Recognition of Familiar Objects?. Perception.

[CR14] Desmarais G, Meade M, Wells T, Nadeau M (2017). Visuo-haptic integration in object identification using novel objects. Atten Percept Psychophys.

[CR15] Elison JT, Wolff JJ, Reznick JS, Botteron KN, Estes AM, Gu H, Hazlett HC, Meadows AJ, Paterson SJ, Zwaigenbaum L, Piven J (2014). Repetitive behavior in 12-Month-Olds later classified with Autism Spectrum Disorder. J Am Acad Child Adolesc Psychiatry.

[CR16] Espenhahn S, Godfrey KJ, Kaur S, McMorris C, Murias K, Tommerdahl M, Bray S, Harris AD (2023). Atypical Tactile Perception in Early Childhood Autism. J Autism Dev Disord.

[CR17] Estes A, Zwaigenbaum L, Gu H, St. John T, Paterson S, Elison JT, Hazlett H, Botteron K, Dager SR, Schultz RT, Kostopoulos P, Evans A, Dawson G, Eliason J, Alvarez S, Piven J (2015). Behavioral, cognitive, and adaptive development in infants with autism spectrum disorder in the first 2 years of life. J Neurodevelopmental Disorders.

[CR18] Frith U (1989). Autism: explaining the enigma.

[CR19] Germani T, Zwaigenbaum L, Bryson S, Brian J, Smith I, Roberts W, Szatmari P, Roncadin C, Sacrey LAR, Garon N, Vaillancourt T (2014). Brief report: Assessment of early sensory Processing in infants at High-Risk of Autism Spectrum Disorder. J Autism Dev Disord.

[CR20] Giannopulu I, Cusin F, Escolano S, Dellatolas G (2008). Cognitive associations of bimanual haptico-visual recognition in preschoolers. Child Neuropsychol.

[CR21] Gori M, Del Viva M, Sandini G, Burr DC (2008). Young Children do not integrate visual and haptic form information. Curr Biol.

[CR22] Gori M, Del Viva M, Sandini G, Burr DC (2008). Young Children do not integrate visual and haptic form information. Curr Biol.

[CR23] Gori M, Sciutti A, Torazza D, Campus C, Bollini A (2024). The effect of visuo-haptic exploration on the development of the geometric cross-sectioning ability. J Exp Child Psychol.

[CR24] Huang Q, Pereira AC, Velthuis H, Wong NML, Ellis CL, Ponteduro FM, Dimitrov M, Kowalewski L, Lythgoe DJ, Rotaru D, Edden RAE, Leonard A, Ivin G, Ahmad J, Pretzsch CM, Daly E, Murphy DGM, McAlonan GM (2022) GABA B receptor modulation of visual sensory processing in adults with and without autism spectrum disorder. Sci Transl Med 14(626). 10.1126/scitranslmed.abg7859 10.1126/scitranslmed.abg7859 34985973

[CR25] Jao RJ, James TW, James KH (2015). Crossmodal enhancement in the LOC for visuohaptic object recognition over development. Neuropsychologia.

[CR26] Joanne Jao R, James TW, James H (2014). Multisensory convergence of visual and haptic object preference across development. Neuropsychologia.

[CR27] Jovanovic B, Drewing K (2014) The influence of intersensory discrepancy on visuo-haptic integration is similar in 6-year-old children and adults. Front Psychol 5(JAN). 10.3389/fpsyg.2014.00057 10.3389/fpsyg.2014.00057 PMC3906500 24523712

[CR28] Kalagher H, Jones SS (2011). Developmental change in young children’s use of haptic information in a visual task: the role of hand movements. J Exp Child Psychol.

[CR29] Kaur M, Srinivasan SM, Bhat AN (2015) Atypical object exploration in infants at-risk for autism during the first year of lifer. *Frontiers in Psychology*, *6*. 10.3389/fpsyg.2015.00798 10.3389/fpsyg.2015.00798 PMC4468838 26136702

[CR30] Kozou H, Azouz HG, Abdou RM, Shaltout A (2018). Evaluation and remediation of central auditory processing disorders in children with autism spectrum disorders. Int J Pediatr Otorhinolaryngol.

[CR31] Lacey S, Campbell C (2006). Mental representation in visual/haptic crossmodal memory: evidence from interference effects. Q J Experimental Psychol.

[CR32] Lacey S, Lin JB, Sathian K (2011). Object and spatial imagery dimensions in visuo-haptic representations. Exp Brain Res.

[CR33] Leekam SR, Nieto C, Libby SJ, Wing L, Gould J (2007). Describing the sensory abnormalities of children and adults with autism. J Autism Dev Disord.

[CR34] Lewis JD, Evans AC, Pruett JR, Botteron KN, McKinstry RC, Zwaigenbaum L, Estes AM, Collins DL, Kostopoulos P, Gerig G, Dager SR, Paterson S, Schultz RT, Styner MA, Hazlett HC, Piven J, Piven J, Hazlett HC, Chappell C, Gu H (2017). The emergence of Network inefficiencies in infants with Autism Spectrum Disorder. Biol Psychiatry.

[CR35] Maenner MJ, Warren Z, Williams AR, Amoakohene E, Bakian AV, Bilder DA, Durkin MS, Fitzgerald RT, Furnier SM, Hughes MM, Ladd-Acosta CM, McArthur D, Pas ET, Salinas A, Vehorn A, Williams S, Esler A, Grzybowski A, Hall-Lande J, Shaw KA (2023). Prevalence and characteristics of Autism Spectrum Disorder among children aged 8 years — Autism and Developmental Disabilities Monitoring Network, 11 sites, United States, 2020. MMWR Surveillance Summaries.

[CR36] Mansour Y, Burchell A, Kulesza RJ (2021). Central auditory and vestibular dysfunction are key features of Autism Spectrum Disorder. Front Integr Nuerosci.

[CR37] Martinovic J, Lawson R, Craddock M (2012) Time Course of Information Processing in Visual and Haptic object classification. Front Hum Neurosci 6. 10.3389/fnhum.2012.00049 10.3389/fnhum.2012.00049 PMC3311268 22470327

[CR42] Miguel O, Sampaio H, Martínez-Regueiro A, Gómez-Guerrero R, López-Dóriga L, Gómez CG, Carracedo S, Fernández-Prieto M (2017). Touch Processing and Social Behavior in ASD. J Autism Dev Disord.

[CR38] Miller M, Sun S, Iosif A-M, Young GS, Belding A, Tubbs A, Ozonoff S (2021). Repetitive behavior with objects in infants developing autism predicts diagnosis and later social behavior as early as 9 months. J Abnorm Psychol.

[CR39] Morrongiello BA, Humphrey GK, Timney B, Choi J, Rocca PT (1994). Tactual Object Exploration and Recognition in blind and sighted children. Perception.

[CR40] Muratori F, Tonacci A, Billeci L, Catalucci T, Igliozzi R, Calderoni S, Narzisi A (2017). Olfactory Processing in Male Children with Autism: atypical odor threshold and identification. J Autism Dev Disord.

[CR41] Nakano T, Kato N, Kitazawa S (2012). Superior haptic-to-visual shape matching in autism spectrum disorders. Neuropsychologia.

[CR43] Ozonoff S, Macari S, Young GS, Goldring S, Thompson M, Rogers SJ (2008). Atypical object exploration at 12 months of age is associated with autism in a prospective sample. Autism.

[CR44] Perrault TJ, Rowland BA, Stein BE (2012) *The Organization and Plasticity of Multisensory Integration in the Midbrain* 22593882

[CR45] Poole D, Poliakoff E, Gowen E, Couth S, Champion RA, Warren PA (2017). Similarities in autistic and neurotypical visual-haptic perception when making judgements about conflicting sensory stimuli. Multisensory Res.

[CR46] Posar A, Visconti P (2022). Early Motor signs in Autism Spectrum Disorder. Children.

[CR49] Purpura G, Costanzo V, Chericoni N, Puopolo M, Luisa Scattoni M, Muratori F, Apicella F (2017). Bilateral patterns of repetitive movements in 6- to 12-Month-Old infants with Autism Spectrum disorders. Front Psychol.

[CR48] Purpura G, Cioni G, Tinelli F (2018) Development of visuo-haptic transfer for object recognition in typical preschool and school-aged children. Child Neuropsychol 24(5). 10.1080/09297049.2017.1316974 10.1080/09297049.2017.1316974 28427295

[CR51] Purpura G, Perazza S, Cioni G, Tinelli F (2019) Visuo-haptic transfer for object recognition in children with periventricular leukomalacia and bilateral cerebral palsy. Child Neuropsychol 25(8). 10.1080/09297049.2019.1602599 10.1080/09297049.2019.1602599 31017037

[CR50] Purpura G, Febbrini Del Magro E, Caputo R, Cioni G, Tinelli F (2021) Visuo-haptic transfer for object recognition in children with peripheral visual impairment. Vision Res 178. 10.1016/j.visres.2020.06.008 10.1016/j.visres.2020.06.008 33070030

[CR47] Purpura G, Cerroni F, Carotenuto M, Nacinovich R, Tagliabue L (2022) Behavioural differences in Sensorimotor profiles: a comparison of preschool-aged children with sensory Processing Disorder and Autism Spectrum disorders. Children 9(3). 10.3390/children9030408 10.3390/children9030408 PMC8947260 35327780

[CR52] Puts NAJ, Wodka EL, Tommerdahl M, Mostofsky SH, Edden RAE (2014). Impaired tactile processing in children with autism spectrum disorder. J Neurophysiol.

[CR53] Riva V, Riboldi EM, Dondena C, Piazza C, Molteni M, Cantiani C (2022). Atypical ERP responses to audiovisual speech integration and sensory responsiveness in infants at risk for autism spectrum disorder. Infancy.

[CR54] Rogers SJ, Hepburn S, Wehner E (2003) Parent Reports of Sensory Symptoms in Toddlers with Autism and Those with Other Developmental Disorders. In *Journal of Autism and Developmental Disorders* (Vol. 33, Issue 6) 10.1023/b:jadd.0000006000.38991.a7 14714932

[CR55] Ropar D, Greenfield K, Smith AD, Carey M, Newport R (2018). Body representation difficulties in children and adolescents with autism may be due to delayed development of visuo-tactile temporal binding. Dev Cogn Neurosci.

[CR56] Russo NM, Skoe E, Trommer B, Nicol T, Zecker S, Bradlow A, Kraus N (2008). Deficient brainstem encoding of pitch in children with Autism Spectrum disorders. Clin Neurophysiol.

[CR57] Schoen SA, Miller LJ, Brett-Green BA, Nielsen DM (2009). Physiological and behavioral differences in sensory processing: a comparison of children with Autism Spectrum disorder and sensory modulation disorder. Front Integr Nuerosci.

[CR58] Sciutti A, Sandini G (2022). To move or not to move: development of fine-tuning of object motion in Haptic Exploration. IEEE Trans Cogn Dev Syst.

[CR59] Shafer RL, Wang Z, Bartolotti J, Mosconi MW (2021). Visual and somatosensory feedback mechanisms of precision manual motor control in autism spectrum disorder. J Neurodevelopmental Disorders.

[CR60] Simpson K, Adams D, Alston-Knox C, Heussler HS, Keen D (2019). Exploring the sensory profiles of children on the Autism Spectrum using the short sensory Profile-2 (SSP-2). J Autism Dev Disord.

[CR61] Snow JC, Culham JC (2021). The treachery of images: how realism influences brain and behavior. Trends Cogn Sci.

[CR62] Spencer J, O’Brien J, Riggs K, Braddick O, Atkinson J, Wattam-Bell J (2000). Motion processing in autism: evidence for a dorsal stream deficiency. NeuroReport.

[CR63] Stein BE, Stanford TR, Rowland BA (2014). Development of multisensory integration from the perspective of the individual neuron. Nat Reviews Neurosci (Vol.

[CR64] Stein BE, Stanford TR, Rowland BA (2020). Multisensory integration and the society for neuroscience: then and now. J Neurosci (Vol.

[CR65] Tavassoli T, Bellesheim K, Tommerdahl M, Holden JM, Kolevzon A, Buxbaum JD (2016). Altered tactile processing in children with autism spectrum disorder. Autism Res.

[CR66] Ting L (2013). Sensory processing and motor skill performance in elementary school children with autism spectrum disorder. Percept Mot Skills.

[CR67] Turi M, Karaminis T, Pellicano E, Burr D (2016). No rapid audiovisual recalibration in adults on the autism spectrum. Sci Rep.

[CR68] Valagussa G, Purpura G, Nale A, Pirovano R, Mazzucchelli M, Grossi E, Perin C (2022). Sensory Profile of children and adolescents with Autism Spectrum Disorder and Tip-Toe Behavior: results of an Observational Pilot Study. Children.

[CR69] Volkmar FR, McPartland JC (2014). From kanner to DSM-5: Autism as an evolving diagnostic concept. Ann Rev Clin Psychol.

[CR70] Wada M, Ikeda H, Kumagaya S (2021). Atypical effects of visual interference on tactile temporal order judgment in individuals with autism spectrum disorder. Multisensory Res.

